# Systematic Evaluation of Reaction Phase Effects on Photocatalytic CO_2_ Reduction Using Cu‐Doped SrTiO_3_

**DOI:** 10.1002/gch2.70145

**Published:** 2026-08-30

**Authors:** Mohammed A. M. Bajiri, Niqab Khan, Julio Cesar Camilo Albornoz Diaz, Gabriel Natulini Vieira, Yara Jaqueline Kerber Araújo, Lucas D. Paquini, Abdo Hezam, Fabio H. B. Lima, Heberton Wender, Renato V. Gonçalves

**Affiliations:** ^1^ São Carlos Institute of Physics University of São Paulo (USP) São Carlos São Paulo Brazil; ^2^ São Carlos Institute of Chemistry (IQSC) University of São Paulo (USP) São Carlos São Paulo Brazil; ^3^ Chair of Industrial Chemistry and Heterogeneous Catalysis Department of Chemistry Technical University of Munich Garching Germany; ^4^ Institute of Physics Federal University of Mato Grosso Do Sul Campo Grande Mato Grosso do Sul Brazil

**Keywords:** CO_2_ reduction, liquid phase, pressurised solid–gas phase reactions, solid–gas phase

## Abstract

Carbon dioxide conversion into value‐added products offers a promising strategy to mitigate greenhouse gas emissions while enabling sustainable fuel production. Among the available approaches, photocatalytic CO_2_ reduction has attracted significant attention, particularly when combined with advanced catalyst design and reaction engineering. In this work, Cu‐doped SrTiO_3_ is employed as a model photocatalyst to systematically investigate the influence of reaction phase on CO_2_ reduction performance across three distinct reaction configurations: solid–liquid (SL), solid–gas (SG), and pressurized solid–gas (PSG). The results demonstrate that the reaction phase strongly affects both activity and product selectivity. The SG configuration exhibits the highest performance, reaching accumulated yields of 11.13 µmol g^−1^ of CH_4_ and 2.32 µmol g^−1^ of CO after 4 h of reaction, owing to enhanced reactant accessibility and reduced mass‐transfer limitations. In contrast, the SL configuration shows the lowest gaseous‐product activity due to limited CO_2_ solubility and accumulation of dissolved intermediates, while favoring the formation of oxygenated liquid products such as methanol and ethanol. The PSG system displays intermediate performance, where increased CO_2_ availability is partially offset by hindered product desorption. These findings highlight reaction‐phase engineering as a key factor governing photocatalytic CO_2_ conversion efficiency and selectivity.

## Graphical abstract

How much does the reaction environment matter in photocatalytic CO_2_ reduction? Using Cu‐doped SrTiO_3_ as a model catalyst, the solid–gas configuration is found to outperform liquid and pressurized systems, revealing that reaction‐phase engineering plays a decisive role in controlling efficiency and product selectivity.

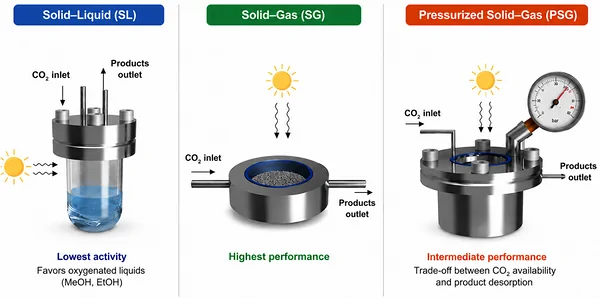

## 1 Introduction

Burning fossil fuels releases large amounts of CO_2_, a major driver of global warming and one of today's most urgent environmental problems [1, 2]. Resolving the ecological issue by capturing CO_2_ and converting it to environmentally friendly energy sources is a great topic of interest. Photocatalytic CO_2_ reduction has attracted much attention because it is a green way to directly convert CO_2_ to other useful chemicals or fuels like CO, HCOOH, CH_3_OH, HCHO, and CH_4_ [3].

Among different developed semiconductors for photocatalytic CO_2_ conversion, SrTiO_3_ is considered a potential candidate due to its unique properties like low toxicity, low cost, and high thermal and photochemical stabilities [4]. However, pristine SrTiO_3_ still exhibits relatively low efficiency, mainly due to its high charge recombination rate, poor CO_2_ activation at the surface, and limited solar spectrum utilization resulting from its wide bandgap (> 3.0 eV) [5]. Thus, further improvements are required to suppress charge recombination and to engineer the bandgap for harvesting a large portion of the solar spectrum. Doping SrTiO_3_ with aliovalent cations has proven effective in modifying the band structure, as it introduces defect levels that contribute to bandgap narrowing [6].

For instance, Cu‐doping of SrTiO_3_ preferentially replaces Ti^4+^ sites with Cu^2+^ or Cu^+^ ions, due to geometric reasons such as ionic radius and coordination number, creating oxygen vacancies and intermediate energy states [7]. These defect levels narrow the effective bandgap, enhancing visible‐light absorption, and act as trapping sites to promote charge separation, thereby reducing electron‐hole recombination [8]. Moreover, Cu dopants improve CO_2_ adsorption and activation at the catalyst surface, providing additional active sites for photocatalytic reduction [9]. Sun et al. demonstrated that Fe, Co, Ni, and Cu‐doped SrTiO_3_@TiO_2_ prepared via hydrothermal synthesis broadened the photoresponse into the visible region, with improved CO_2_ reduction in water to selectively yield C_1_ products, attributed to Ti^3+^ species formed by charge compensation [10]. Ding et al. showed that controlling the Cu^+^/Cu^2+^ and Cu^+^/Cu^0^ ratios in Cu‐doped SrTiO_3_ nanofibers modulates methanol selectivity, with a linear correlation between the Cu^+^/Cu^2+^ ratio and methanol generation rate [9]. Similarly, Li et al. reported that slight Cu^2+^ doping at the B‐site of SrTiO_3_ forms stable CuO_6_ octahedra, enhances charge transfer, and tunes the electronic structure, resulting in more efficient CO_2_ adsorption and over an order‐of‐magnitude improvement in CO_2_ electroreduction activity and selectivity [11].

While these studies demonstrate the intrinsic benefits of Cu‐doping to tailor the electronic structure and surface reactivity of SrTiO_3_‐based photocatalysts, the reaction environment is equally critical for improving performance in CO_2_ reduction. In fact, many reaction environments like solid–liquid (SL) and solid–gas (SG) phases, including under pressurized CO_2_, have been adopted to effectively reduce CO_2_ to hydrocarbon fuels. The SL configuration has shown limited CO_2_ solubility, reduction competitions of CO_2_ and water molecules, and preventing efficient interaction between the catalyst and CO_2_ [12]. However, the SG configuration reactors provide advantages like enhanced reactant mixing and regulation of the CO_2_/H_2_O ratio, resulting in superior CO_2_ adsorption on the catalyst surface [13, 14]. In addition, the reaction phase (SG, SL, or SG under pressure) substantially affects the production rates of the hydrocarbons.

This work highlights the influence of the reaction phase on the photocatalytic CO_2_ reduction capability of Cu‐doped SrTiO_3_. Our findings demonstrate that the liquid phase exhibited the lowest activity, while the SG with pressure phase showed comparable activity to the SG phase. These differences could be attributed to the formation of dissolved products in the liquid phase and the slightly more difficult desorption caused by increased surface pressure in the SG with pressure phase. These findings demonstrate the significance of suitable reaction conditions to enhance the efficacy of photocatalytic CO_2_ reduction.

## 2 Results and Discussion

### 2.1 Structural and Morphological Studies

In this work, we employ our previously optimized 2%Cu‐doped SrTiO_3_ (Cu:STO) sample [15] as a benchmark material to systematically investigate the influence of different reactor configurations on photocatalytic performance. By using this well‐characterized and high‐performing material, we ensure that any observed differences arise primarily from the reaction environment rather than from variations in material properties.

**FIGURE 1:**
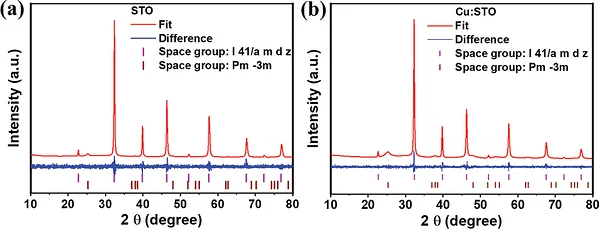
X‐ray diffraction (XRD) patterns with Rietveld refinement. (a) STO and (b) Cu:STO show the ratio between the SrTiO_3_ and TiO_2_ phases in each sample, along with their respective space groups.

The crystal structures of the STO and Cu:STO samples were confirmed by X‐ray diffraction (XRD) analysis, revealing differences in their crystal structures after the Cu‐doping. Figure 1a,b exhibits representative XRD patterns of STO and Cu:STO samples, respectively. The XRD patterns confirmed the presence of SrTiO_3_ as the main crystalline phase along with TiO_2_ as a secondary phase in both undoped STO and Cu:STO. The refinements of the crystalline structure of pure STO and Cu:STO using the Rietveld method were performed assuming substitutional doping of Cu into the structure of the STO in the position of Ti, and with a secondary phase of TiO_2_ [15]. The refinement indicated that the TiO_2_ fraction was approximately 3.2% in STO and increased to 11.1% in Cu:STO. The detailed Rietveld refinement parameters for STO and Cu:STO are summarized in Table S1. The diffraction peaks were indexed using standard data from the ICSD card no. 19796 for STO and no. 121632 for TiO_2_, which served as references for Rietveld refinement. The presence of TiO_2_ is expected, likely resulting from a reaction between titanium isopropoxide and atmospheric moisture or water [16, 17]. Consequently, the addition of Cu(CO_2_CH_3_)_2_.H_2_O, an essential material for copper doping, directly introduces water into the reaction, which increases the fluidity needed for TiO_2_ formation. Furthermore, the increased TiO_2_ content in Cu:STO resulting from the Cu‐doping process may indicate phase segregation or the formation of secondary phases [18]. These structural changes are also reflected in the morphology of the material, as SEM images (Figures S1 and S2) reveal a noticeable reduction in particle size after Cu incorporation, suggesting that the dopant influences both phase evolution and particle growth during synthesis.

### 2.2 Photocatalytic CO_2_ Reduction

**FIGURE 2:**
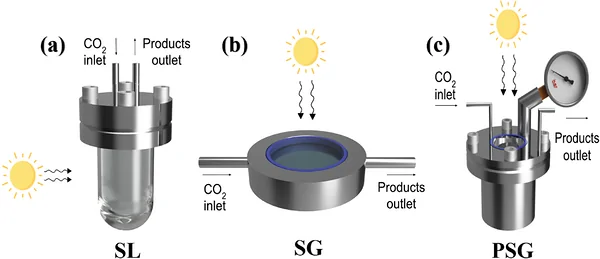
Schematic illustration of the photocatalytic reactors used in this study: (a) solid–liquid (SL) phase using a quartz reactor with catalyst suspension, (b) solid–gas (SG) phase with catalyst exposed to a flowing CO_2_ gas stream, and (c) pressurized solid–gas (PSG) phase reactor operated under controlled pressure (up to 3 bar). Arrows indicate CO_2_ inlet and product outlet, while illumination is provided by a Xenon Lamp at 500 mW.cm^−2^ through a quartz window.

The photocatalytic CO_2_ reduction performance of Cu:STO was evaluated under three distinct interfacial configurations: SL, SG, and pressurized solid–gas (PSG) phases, as illustrated in Figure 2. In all experiments, identical catalyst loading, irradiation conditions, and analytical procedures were employed to ensure reliable comparison between the different reactor configurations. Significant differences in activity and selectivity were observed across the three configurations.

**FIGURE 3:**
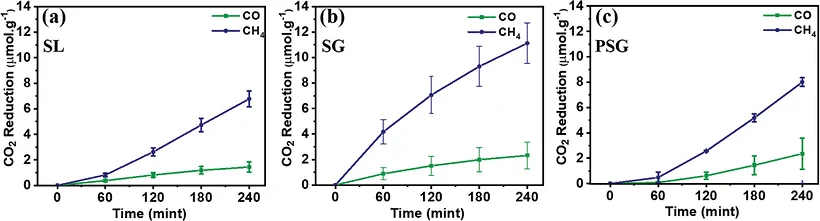
Photocatalytic CO_2_ reduction of Cu:STO with (a) SL, (b) SG, and (c) PSG configurations. The error bars are the standard deviation of three independent measurements.

Figure 3 summarizes the photocatalytic performance of the Cu:STO sample under three different reaction environments: SL, SG, and PSG. In the SL configuration (Figure 3a), the photocatalytic activity was the lowest among the three systems, yielding 1.3 µmol g^−1^ of CO and 6.3 µmol g^−1^ of CH_4_ after 240 min of reaction. This reduced performance can be attributed to the limited solubility of CO_2_ in water, which restricts its availability at the catalyst surface [19]. Additionally, competitive adsorption of water molecules and the accumulation of dissolved intermediates hinder efficient charge transfer and product desorption. These factors not only suppress the formation of gaseous products but also favor the generation of liquid‐phase products such as methanol and ethanol.

The SG configuration (Figure 3b) exhibited the highest photocatalytic performance, yielding 11.1 µmol g^−1^ of CH_4_ and 2.3 µmol g^−1^ of CO after 240 min. This enhanced activity can be attributed to the direct gas‐solid interaction, which maximizes CO_2_ accessibility at the catalyst surface and promotes more efficient utilization of active sites. Unlike SL systems, the absence of a solvent eliminates diffusion limitations associated with CO_2_ dissolution and transport, enabling faster mass transfer and more effective reactant delivery [20]. Furthermore, the SG configuration environment reduces competitive adsorption from solvent molecules and suppresses side reactions, allowing for more efficient charge transfer and reaction pathways toward hydrocarbon formation [20, 21, 22]. The improved interfacial conditions also facilitate rapid desorption of products, preventing site blocking and sustaining catalytic turnover. As a result, the SG configuration not only enhances overall activity but also improves selectivity toward CH_4_, highlighting the critical role of the reaction interface in governing photocatalytic CO_2_ reduction.

The PSG configuration (Figure 3c) exhibited intermediate photocatalytic performance compared to the SL and SG systems. Increasing the CO_2_ pressure to 3 bar enhances the availability of CO_2_ at the catalyst surface, which can promote adsorption and potentially facilitate its activation [23]. However, this increased surface coverage also alters the adsorption‐desorption equilibrium, leading to significant changes in interfacial reaction dynamics. In particular, the higher CO_2_ pressure may stabilize surface‐bound intermediates and products, making their desorption more difficult. This can result in partial blockage of active sites, thereby limiting the continuous turnover of the catalytic cycle. Consequently, although reactant availability is improved under pressurized conditions, the restricted product desorption and modified surface equilibria can offset these benefits, preventing further enhancement in overall activity compared to the SG configuration [24]. Thus, excessive CO_2_ pressure in our system, while beneficial for increasing reactant concentration, may negatively impact catalytic performance by hindering surface regeneration and altering reaction pathways.

Overall, the SG configuration demonstrated superior efficiency, producing approximately twice as much as the SL and 1.5 times more than the PSG configuration. This system also showed particularly high selectivity toward CH_4_ under anaerobic conditions. These findings highlight the importance of reactor design and reaction conditions in determining product distribution and efficiency in photocatalytic CO_2_ reduction.

**FIGURE 4:**
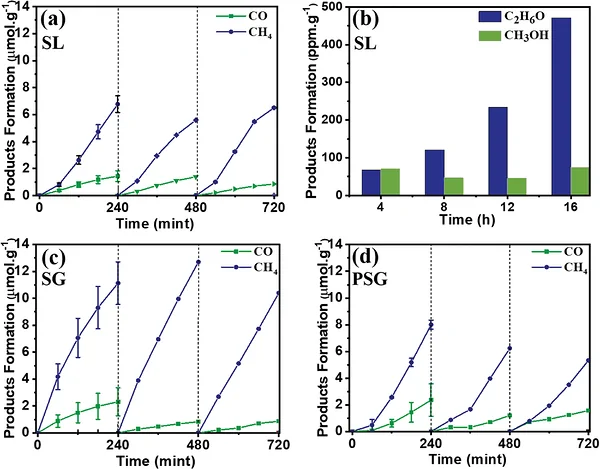
Recyclability of Cu:STO with different phases under each configuration: (a) SL (gas products), (b) SL (liquid products), (c) SG, and (d) PSG configuration. The error bars are the standard deviation of three independent measurements.

The CO_2_ photoreduction stability of the Cu:STO was evaluated over three consecutive cycles under all reaction configurations (Figure 4). In the SL configuration, the evolution of gaseous products (CO and CH_4_) is shown in Figure 4a, where both products exhibit stable production over three 4‐h cycles, indicating good catalytic stability under these conditions. However, the overall yield of gaseous products remains relatively low, which can be attributed to the limited solubility of CO_2_ in water, restricting its availability at the catalyst surface and thereby suppressing gas‐phase product formation. Instead, this behavior is primarily driven by the solubility of CO_2_ and reaction intermediates in the liquid medium, which enhances their residence time at the catalyst surface and enables further transformation into C_1_ and C_2_ products.

In parallel, analysis of the liquid product of the SL configuration (Figure 4b) reveals the formation of oxygenated products, specifically methanol (CH_3_OH) and ethanol (C_2_H_5_OH), after prolonged reaction times (up to 16 h). Methanol reaches a maximum concentration of approximately 70.8 ppmmol g^−1^ (66.33 µmol g^−1^) within the first 4 h and subsequently decreases, whereas ethanol shows a continuous increase, reaching ∼500 µmol g^−1^ after 16 h. This opposite trend suggests a sequential reaction pathway, where C_1_ intermediates are progressively converted into C_2_ products via C─C coupling reactions on the catalyst surface. The observed behavior indicates that methanol likely acts as an intermediate, undergoing further transformation into ethanol under irradiation [25, 26]. Such pathways are consistent with the accumulation of dissolved intermediates in the liquid phase, which facilitates secondary reactions that are less favorable in gas‐phase systems. Moreover, the presence of water promotes alternative reaction routes involving proton‐coupled electron transfer, favoring the formation of oxygenated products over hydrocarbons. Thus, these results demonstrate that the SL configuration promotes the formation of liquid‐phase products (CH_3_OH and C_2_H_5_OH), while limiting the evolution of gaseous species (CO and CH_4_). This behavior is primarily driven by the solubility of CO_2_ and reaction intermediates in the liquid medium, which enhances their residence time at the catalyst surface and enables further transformation into C_1_ and C_2_ products.

To confirm this selectivity shift, an electron‐equivalent balance was calculated after 4 h of reaction. Gas products (CO and CH_4_) accounted for only 5.4% (53.0 µmol e^−^ g^−1^) of the consumed photogenerated electrons, while the reliably quantified liquid products (methanol and ethanol) accounted for 94.6% (928.50 µmol e^−^ g^−1^) (calculation details in Supporting Information) [27]. This demonstrates that the catalytic activity in the SL phase is not inherently poor, but is overwhelmingly directed toward liquid products.

According to existing literature, the reduction of SL CO_2_ is assumed to occur through direct sequential surface processes. Contemporary mechanistic investigations indicate that methanol and methane function as competitive, parallel products originating from common surface intermediates, rather than through subsequent partial oxidation of methane [28]. Simultaneously, the CO_2_ reduction produces essential C1 intermediates like formate (^*^HCOO), formaldehyde (^*^CH_2_O), and methoxy groups (^*^CH_3_O). Moreover, the synthesis of multi‐carbon compounds or ethers can transpire through the surface coupling of these C1 fragments. As indicated, the interaction of methoxy intermediates (^*^CH_3_O) with formaldehyde (^*^CH_2_O) offers a feasible route to produce dimethyl ether (CH_3_OCH_3_) and H_2_O [26, 29].

The stability tests performed under SG and PSG configurations (Figure 4c,d) demonstrate sustained photocatalytic activity over multiple reaction cycles, confirming the robustness of the Cu:STO catalyst under gas‐phase conditions. In both systems, CH_4_ production remains dominant, while CO selectivity is either slightly reduced or remains nearly constant, indicating a preferential reaction pathway toward methane formation. Notably, in the SG configuration (Figure 4c), an increase in CH_4_ production is observed during the second cycle, which may be attributed to a surface activation process. Such behavior has been previously reported and is often associated with the formation of additional active sites, improved charge separation, or the stabilization of catalytically favorable surface states during initial operation [30, 31].

The PSG system (Figure 4d) also exhibits stable performance, although without a pronounced activation effect, suggesting that elevated pressure conditions may limit dynamic surface restructuring compared to ambient SG conditions. Additionally, this incremental activation is absent in the SL phase (Figure 4a), where fast adsorption competition by liquid water creates an immediate, though kinetically limited, steady‐state surface coverage. Overall, these results demonstrate that Cu:STO maintains high stability and catalytic efficiency across all reaction environments (SL, SG, and PSG), without significant structural or morphological modification, as further supported by post‐reaction characterization (Figure 6). This highlights the suitability of this material for sustained photocatalytic CO_2_ reduction under varying operational conditions.

**FIGURE 5:**
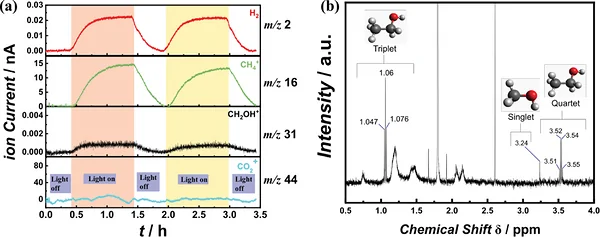
(a) Time‐resolved online mass spectrometry (MS) signals of the gaseous products and volatilized methanol during alternating light ON/OFF cycles. (b) ^1^H‐NMR spectrum of the liquid‐phase products following 4 h of photocatalytic CO_2_ reduction in the SL configuration.

Multiple independent methods were used to further evaluate the dynamic stability of the Cu:STO catalyst and to confirm the origin of the carbon products. First, the identification ruled out adventitious carbon contamination and evaluated the operational stability of the photocatalytic process and production by time‐resolved online mass spectrometry (MS) was conducted under a continuous CO_2_ flow (20 mL min^−1^). The system was subjected to alternating light ON/OFF cycles to monitor the real‐time generation of volatile species. As depicted in Figure 5a, the generation of characteristic molecular fragments, including *m/z* = 16 (CH_4_ ion molecular) and *m/z* = 26 corresponding to C_2_ species, such as C_2_H_4_ (see Figure S3 and Reference [15]), exhibited a reversible dependence on UV–vis irradiation. The online MS detected the volatilized methanol at *m/z* = 31 (CH_2_OH^+^), even though its vapor‐phase concentration was near the detection limit of the instrument compared to ethanol. Concurrently, the CO_2_ signal (*m/z* = 44) is presented as a constant, straight baseline, as it acts as the continuous carrier and reactant gas for the reduction process (Figure 5a and Figure S3). Upon illumination, the ion currents for these product species rise sharply with the same pattern, and they decay rapidly when the light source is turned off.

Furthermore, 1H‐NMR spectroscopy was used to clearly confirm the liquid‐phase oxygenates produced in the SL configuration. The obtained spectrum exhibits the distinctive triplet and quartet splitting patterns that verify the presence of ethanol in addition to the typical singlet resonance associated with methanol, as seen in Figure 5b. The quantitative GC‐FID analysis is fully supported by this independent structural confirmation. The Cu:STO catalyst's exceptional photo‐stability and resistance to deactivation are demonstrated by its ability to consistently switch on product generation over repeated illumination cycles under a continuous flowing gas stream. More significantly, this dynamic, light‐responsive behavior unequivocally demonstrates that the hydrocarbons and oxygenates found are the result of ongoing, light‐driven photocatalytic reduction of the supplied CO_2_. Instead of sustained, cyclical regeneration precisely timed with the photon flux, the product evolution would show a continuous decay profile if they were produced by the breakdown of leftover organic contaminants on the catalyst surface.

**FIGURE 6:**
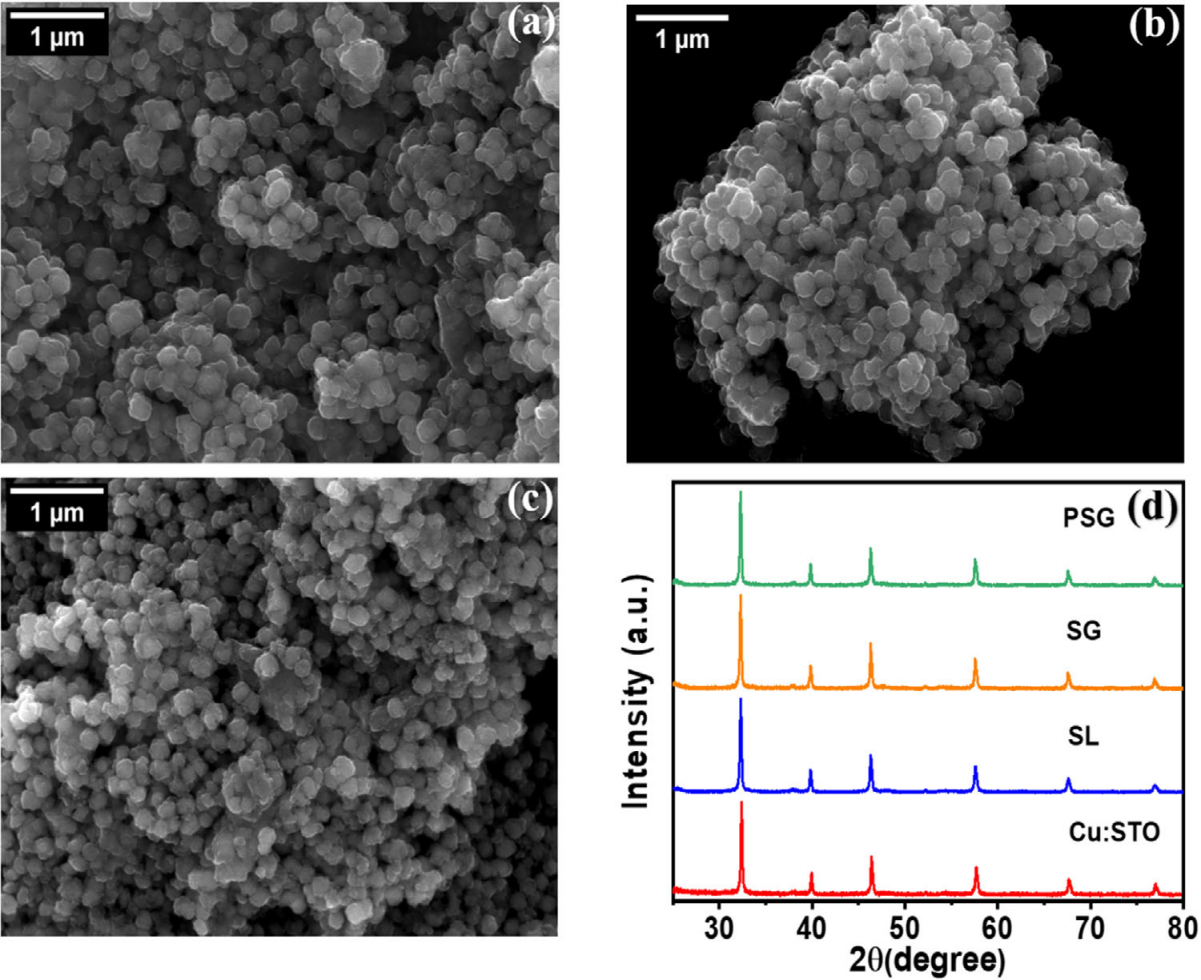
FE‐SEM of Cu:STO after the recyclability of different reaction phases (a) SL, (b) SG, and (c) PSG. (d) The XRD after the recyclability test of Cu:STO with different reaction phases.

**FIGURE 7:**
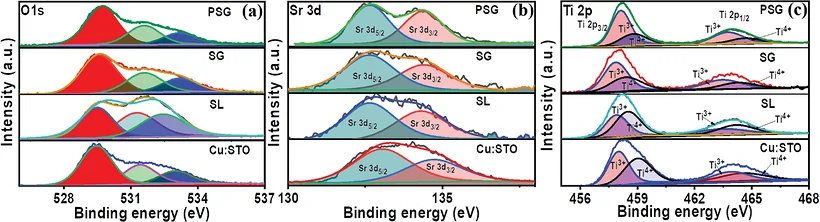
The XPS of Cu:STO and after the recyclability test with different phases: (a) O1s, (b) Sr 3d, and (c) Ti 2p. PSG: XPS after the recyclability test for a SG reaction under 3 bar of CO_2_ pressure. SG: XPS after the recyclability test for a SG reaction at ambient pressure. SL: XPS after the recyclability test for a liquid‐phase reaction.

Post‐reaction SEM, XRD, and XPS analyses were carried out to further clarify the catalyst's structural and electronic stability (Figures 6 and 7). These findings imply that the Cu:STO is extremely stable regardless of the reaction phase, as is the stable morphology and structure seen in all phases (SL, SG, and PSG). Additionally, the Cu:STO's structural integrity is confirmed by the post‐stability XPS analysis, which offers a clear understanding of the elemental composition.

The O 1s spectra (Figure 7a) show no significant shift in binding energy for the SG and PSG configurations, indicating that the oxygen environment remains largely unchanged under gas‐phase conditions. In contrast, the SL configuration exhibits noticeable variations in the relative intensity of hydroxyl groups and adsorbed oxygen species, reflecting the strong interaction between the catalyst surface and the aqueous environment. The Sr 3d spectra (Figure 7b) remain essentially unchanged across all configurations, confirming the stability of the SrTiO_3_ framework. Considering the photocatalytic activities of the SL and SG systems, the PSG configuration, as shown in Figure 3c, has an intermediate photocatalytic activity. While increasing the CO_2_ pressure to 3 bar enhances the initial availability of CO_2_ at the catalyst surface, it creates a kinetic bottleneck through the excessive surface stabilization of intermediates. As indicated by post‐reaction XPS analysis (Figure 6c, Table S2), the PSG system exhibits the highest Ti^3+^ content (Ti^3+^/Ti^4+^ ∼2.3), followed by the SG configuration (∼1.6) and the SL phase (∼0.81). This demonstrates that gas‐phase conditions, particularly under elevated CO_2_ pressure, promote the reduction of Ti^4+^ to Ti^3+^ through strong CO_2_ adsorption and surface reconstruction processes, where the formation of surface carbonate‐like species induces charge transfer from the lattice to maintain charge balance [32]. Importantly, the correlation between the Ti^3+^/Ti^4+^ ratio and catalytic activity is non‐linear. The PSG system possesses ∼44% more Ti^3+^ than the SG system, yet it produces significantly less CH_4_ (∼8.0 µmol g^−1^ compared to 11.1 µmol g^−1^). This inverse relationship‐where a higher Ti^3+^ concentration results in a lower CH_4_ yield‐clearly indicates that the reaction is limited by product desorption. Specifically, the elevated pressure induces excessive Ti^3+^ concentrations, which lead to overly strong binding of reaction intermediates. Because these intermediates are bound too tightly, they fail to desorb efficiently, resulting in the blockage of active sites. This desorption bottleneck restricts the continuous turnover of the catalytic cycle, ultimately offsetting the kinetic benefits of increased reactant availability. This interpretation perfectly aligns with the Sabatier principle, which dictates that optimal catalytic performance requires balanced intermediate binding‐neither too weak to cause poor activation nor too strong to hinder product desorption [33]. Similar volcano relationships driven by adsorption strength have been recently demonstrated in gas‐phase photocatalytic systems, such as the Ti^3+^/Ti^4+^ containing TiO_2_/Ag_2_S nanowires, was reported that optimal defect‐driven dynamics were shown to govern CO_2_ to CH_4_ conversion efficiency [34].

More importantly, the Ti 2p spectra (Figure 7c) reveal pronounced differences in the Ti^3+^/Ti^4+^ ratio depending on the reaction phase. The PSG system shows the highest Ti^3+^ content (Ti^3+^/Ti^4+^ ≈ 2.3), followed by the SG configuration (≈1.6), while the SL phase exhibits the lowest fraction (≈0.813). This trend indicates that gas‐phase conditions, particularly under elevated CO_2_ pressure, promote the reduction of Ti^4+^ to Ti^3+^, leading to enhanced surface electron density and modified catalytic properties. The increased Ti^3+^ content under PSG conditions can be attributed to strong CO_2_ adsorption and surface reconstruction processes. Under high CO_2_ pressure, the formation of surface carbonate‐like species induces charge transfer from the lattice, reducing Ti^4+^ to Ti^3+^ to maintain charge balance [32]. This process alters the electronic structure and surface reactivity, influencing adsorption strength, intermediate stabilization, and reaction pathways. However, while higher Ti^3+^ concentrations can enhance CO_2_ activation, they may also lead to stronger binding of intermediates, which is consistent with the previously observed limitation in product desorption under PSG conditions.

In contrast, the SL configuration, which favors the formation of methanol and ethanol, exhibits the lowest Ti^3+^ fraction. This suggests that liquid‐phase reactions proceed via a distinct mechanism, likely dominated by hydration‐mediated pathways and proton‐coupled electron transfer processes. The presence of water competes with CO_2_ for adsorption sites and suppresses CO_2_‐induced surface reduction, thereby stabilizing Ti^4+^ states while enabling alternative C‐C coupling pathways for oxygenate formation.

It is worth noting that Cu species were not clearly detected in the XPS spectra, likely due to their low concentration and dispersion below the detection limit [15]. Nevertheless, the observed changes in Ti oxidation states and catalytic behavior strongly support the role of Cu doping in modulating the electronic structure and facilitating charge transfer processes. Overall, the combined catalytic and spectroscopic results demonstrate that Cu:STO maintains structural stability while undergoing phase‐dependent electronic modifications, with the Ti^3+^/Ti^4+^ balance playing a key role in governing activity, selectivity, and reaction pathways across different interfacial environments.

## 3 Conclusion

In this work, we systematically demonstrated that the photocatalytic CO_2_ reduction performance of Cu‐doped SrTiO_3_ is strongly governed by the reaction phase, highlighting the critical role of the catalyst‐reactant interface in determining activity and selectivity. Among the investigated configurations, the SG phase exhibited the highest performance, which can be attributed to enhanced CO_2_ accessibility, efficient mass transport, and the absence of competitive adsorption from solvent molecules. These conditions promote favorable reaction kinetics, efficient charge utilization, and selective formation of gaseous products, particularly CH_4_. In contrast, the SL phase showed lower activity but favored the formation of oxygenated products such as methanol and ethanol. This behavior is associated with limited CO_2_ solubility, competitive adsorption of water, and the accumulation of dissolved intermediates, which enable secondary reactions such as C─C coupling. The PSG configuration exhibited intermediate performance, revealing that although increased CO_2_ pressure enhances surface coverage and adsorption, it also alters interfacial equilibria and can hinder product desorption, thereby limiting catalytic turnover. Post‐reaction XPS analysis provides key insights into the phase‐dependent electronic structure of the catalyst. Gas‐phase conditions, particularly under pressure, promote the formation of Ti^3+^ species through CO_2_‐induced surface reduction and charge redistribution, while the SL phase preserves Ti^4+^ states due to the dominant role of water in surface interactions. These findings establish a clear correlation between the Ti^3+^/Ti^4+^ ratio, reaction environment, and catalytic behavior, emphasizing the importance of electronic structure modulation in photocatalytic CO_2_ conversion. Overall, these findings demonstrate that optimizing the reaction environment is as critical as catalyst design for achieving high‐performance photocatalytic systems.

## 4 Experimental Section

All chemicals and reagents used in this work were of analytical grade and were used as received without further purification.

### 4.1 Synthesis and Characterization of Photocatalysts

The Cu‐doped SrTiO_3_ photocatalyst was synthesized following our previously reported method, with minor modifications [15]. Briefly, 7.6 mL of titanium isopropoxide (Acros, 98%) was dissolved in 25 mL of isopropanol. Subsequently, 0.02 M copper acetate (Cu(CO_2_CH_3_)_2_·H_2_O, (Mallinckrodt, 98.7%) and 1 M strontium nitrate (Sigma–Aldrich, 99%) were added and stirred for 30 min to ensure complete dissolution. The resulting solution was then added dropwise into 15 mL of 5 M aqueous KOH under continuous stirring, followed by an additional 1 h of stirring. The pH was adjusted to 7, and the mixture was transferred to a Teflon‐lined autoclave and heated at 200°C (5°C min^−1^) for 4 h. After synthesis, the product was washed once with acetic acid and multiple times with deionized water to remove residual impurities, and then dried at 80°C overnight. Undoped SrTiO_3_ (STO) was prepared using the same procedure without the addition of a copper precursor. The Cu‐doped SrTiO_3_ sample was denoted as Cu:STO, while the undoped material was referred to as STO. The materials were characterized by X‐ray diffraction (XRD), field‐emission scanning electron microscopy (FESEM), and X‐ray photoelectron spectroscopy (XPS). Additional experimental details are provided in the Supporting Information.

### 4.2 Photocatalytic CO_2_ Reduction Under Different Reaction Phases

The photocatalytic CO_2_ reduction performance of Cu:STO was evaluated under three distinct interfacial configurations: SL, SG, and PSG phases, as illustrated in Figure 2. In all experiments, identical catalyst loading, irradiation conditions, and analytical procedures were employed to ensure reliable comparison between the different reactor configurations.

#### 4.2.1 SL Phase

Photocatalytic reactions in the SL configuration were carried out in a 100 mL quartz reactor. A suspension was prepared by dispersing 50 mg of Cu‐doped SrTiO_3_ in 30 mL of deionized water, followed by ultrasonication for 5 min to ensure homogeneous dispersion. Prior to irradiation, the suspension was purged with high‐purity argon (99.99%) for 10 min, followed by vacuum‐air cycling to remove residual gases. Subsequently, humidified CO_2_ was introduced at a flow rate of 4 mL min^−1^ for 20 min to saturate the system. During photocatalysis, the CO_2_ flow was reduced to 1 mL min^−1^ and maintained throughout the reaction. Illumination was provided by a Xe lamp calibrated to 500 mW cm^−2^ with an AM 1.5 filter. The reaction proceeded for 4 h under continuous irradiation. Gaseous products were periodically analyzed using an online gas chromatograph (Agilent 7890B) equipped with a flame ionization detector (FID) coupled to a nickel‐based methanizer for enhanced detection of CO and CO_2_. The transfer line connecting the reactor to the GC was maintained at 80°C to prevent condensation of reaction products. Separation of CO and CH_4_ was achieved using an HP‐PLOT/Q column, coupled in series with an HP‐MOLESIEVE column for permanent gases.

The quantitative analyses of liquid products were conducted using a GC‐FID system with a liquid autosampler (Agilent 8860, G2790A). 1H‐nuclear magnetic resonance (1D 1H‐NMR) measurements were performed to identify and qualitatively evaluate the formation of liquid products generated during the photocatalytic CO_2_ reduction process, particularly methanol (MeOH) and ethanol (EtOH). Spectra were acquired using an Agilent Technologies 500/54 Premium Shield spectrometer operating at 500 MHz for 1H detection. After the photocatalytic experiments, aliquots of the solution were collected and mixed with deuterated water (D_2_O). All spectra were recorded at room temperature using standard one‐dimensional proton acquisition sequences with water suppression. From that, 1024 scans were accumulated, employing a relaxation delay of 2s and a spectral width sufficient to cover the 2–14 ppm range, PW90: 7.7 µs, and a total procedure time of 1 h 44 min.

#### 4.2.2 SG Phase

The SG experiments were conducted in a custom‐designed stainless‐steel reactor (4 mL) equipped with a quartz optical window to allow irradiation of the catalyst. The photocatalyst (50 mg) was evenly distributed as a thin layer at the bottom of the reactor using a mesh support to maximize exposure to the gas phase. Before illumination, the reactor was purged with humidified CO_2_ at a flow rate of 4 mL min^−1^ for 20 min to remove residual gases and establish a controlled reaction atmosphere. The gas flow was regulated by a mass flow controller (MFC) positioned upstream of the reactor. During the photocatalytic reaction, the CO_2_ flow was reduced to 1 mL min^−1^. The system was irradiated under identical conditions to the SL experiments (Xe lamp, 500 mW cm^−2^, AM 1.5 filter) for 4 h. Product analysis was performed using the same online GC setup and operating conditions described above.

#### 4.2.3 PSG Phase

Photocatalytic CO_2_ reduction under PSG conditions was carried out in a stainless‐steel reactor with a total volume of 60 mL, designed to operate under controlled pressure. The catalyst (50 mg) was uniformly distributed at the bottom of the reactor in a similar manner to the SG configuration. The reactor was initially purged with CO_2_ at 4 mL min^−1^ for 20 min to remove residual gases and ensure CO_2_ saturation. Subsequently, the flow rate was reduced to 1 mL min^−1^, and the system was gradually pressurized up to 3 bar of CO_2_. In this configuration, the mass flow controller was installed downstream of the reactor, enabling regulation of gas and maintenance of internal pressure. The reaction was performed at room temperature under continuous illumination using the same Xe lamp setup (500 mW cm^−2^, AM 1.5 filter) for 4 h. In the SG and PSG configurations, the CO_2_ gas stream was humidified before entering the reactor to provide water as the electron donor. The gaseous products were analyzed using the same gas chromatography setup.

#### 4.2.4 Online MS

Online MS measurements were performed using an OmniStar GSD320 quadrupole mass spectrometer (Pfeiffer Vacuum) equipped with a stainless‐steel capillary inlet (0.125 mm inner diameter). The mass spectrometer was directly coupled to the photochemical reactor through a capillary interface, enabling continuous monitoring of volatile and/or gaseous products generated during photocatalytic CO_2_ reduction. A continuous CO_2_ flow (20 mL min^−1^) was controlled using a flowmeter (AALBORG ‐GFC17A‐VAL6‐A0‐3NC‐10‐SS) and supplied to the reactor to maintain solution saturation and stable mass transport conditions throughout the experiments. An initial exploratory analysis was conducted by acquiring full mass spectra (Δ*m/z*: 0–50 m.a.u). The measurements were taken over separate 2‐h intervals under the solar simulator (500 mW/cm^−2^, using a standard AMG filter), allowing qualitative identification of the volatile species generated during the photocatalytic process. Subsequently, time‐resolved MS experiments were carried out using selected ion monitoring (SIM) mode focused on the characteristic *m/z* molecular ion or fragments of the main CO_2_ reduction products and possible side products. For this case, we performed a stability test with a mass spectrum being initially recorded under dark conditions for 30 min. The solar simulator was then alternately switched on for 1 h and off for 30 min over two consecutive cycles to assess the photocatalyst activity and stability under intermittent UV–vis irradiation, as well as the relaxation behavior of the photochemical system. In this case, selected mass‐to‐charge (*m/z*) signals corresponding to molecular ions and characteristic fragments were continuously monitored, including *m/z* = 2 (H_2_), 15 (CH_3_^+^), 16 (CH_4_^+^), 26 (C_2_H_2_^+^), 31 (CH_2_OH^+^), 32 (CH_3_OH^+^), and 44 (CO_2_^+^).

## Conflicts of Interest

The authors declare no conflicts of interest.

## Supporting information




**Supporting File**: gch270145‐sup‐0001‐SuppMat.docx.

## Data Availability

The data that support the findings of this study are available from the corresponding author upon reasonable request.
