# Supplementary material for: Systematic Evaluation of Reaction Phase Effects on Photocatalytic CO2 Reduction Using Cu‐Doped SrTiO3
Source: Glob Chall. 2026 Aug 30;10(9):e70145. doi: 10.1002/gch2.70145 (PMC13527401; doi:10.1002/gch2.70145)
Supplement: Supplementary file 1 [file GCH2-10-e70145-s001.docx]

Supporting Information

**Systematic Evaluation of Reaction Phase Effects on Photocatalytic CO_2_ Reduction Using Cu-Doped SrTiO_3_**

*Mohammed A M Bajiri^1*^, Niqab khan^1^, Julio Cesar Camilo Albornoz Diaz^1^, Gabriel Natulini Vieira^1^, Yara Jaqueline Kerber Araújo^1^, Lucas D. Paquini*^2^, *Abdo Hezam^3^, Fabio H.B. Lima*^2^, *Heberton Wender^4^, and Renato V. Gonçalves^1*^*

^1^São Carlos Institute of Physics, University of São Paulo (USP), São Carlos 13560-970, SP, Brazil.

^2^ São Carlos Institute of Chemistry (IQSC), University of São Paulo (USP) , São Carlos, 13563-120, SP, Brazil.

^3^Chair of Industrial Chemistry and Heterogeneous Catalysis, Department of Chemistry, Technical University of Munich, Lichtenbergstraße 4, Garching 85748, Germany.

^4^Institute of Physics, Federal University of Mato Grosso do Sul, Av. Costa e Silva S/N, 79070-900, Campo Grande, MS, Brazil

E-mail: E-mail: rgoncalves@ifsc.usp.br and mbajiri@usp.br

**1. Characterization**

## X-ray diffraction (XRD) was used to investigate the structural properties (Rigaku Ultima IV diffractometer) with a Cu Kα radiation with 2θ angle from 10° to 80° and a step scan of 0.02°, the refinement of the XRD data was by GSAS-II software. Scanning electron microscopy (FESEM) was used to investigate the morphology of the samples (Scanning Electron Microscope - Brand: JEOL - Model: JSM 7200F). Surface composition was analyzed by X-ray photoelectron spectroscopy (XPS) using a Scienta-Omicron ESCA+ spectrometer equipped with a monochromatic Al Kα radiation source (hν = 1486.6 eV) and an electron flood gun to compensate for surface charging effects. Spectra were acquired with a pass energy of 30 eV and a step size of 0.05 eV. All binding energies were calibrated using the adventitious carbon C 1s peak at 284.8 eV as a reference. Data processing and peak fitting were carried out using CasaXPS software, applying a Shirley-type background subtraction prior to curve fitting.

**Table S1.** Rietveld refinement results for STO and Cu:STO samples.

| **Sample** | **STO** | | **Cu:STO** | |
| --- | --- | --- | --- | --- |
| **Crystalline pahse** | **SrTiO_3_** | **TiO_2_** | **SrTiO_3_** | **TiO_2_** |
| Phase fraction (%) | 96.8 | 3.2 | 88.9 | 11.1 |
| a (Å) | 3.91922 | 3.78826 | 3.91858 | 3.78867 |
| b (Å) | 3.91922 | 3.78826 | 3.91858 | 3.78867 |
| c (Å) | 3.91922 | 9.48797 | 3.91858 | 9.48768 |
| alpha (°) | 90 | 90 | 90 | 90 |
| beta (°) | 90 | 90 | 90 | 90 |
| gamma (°) | 90 | 90 | 90 | 90 |
| Cell Volume (Å^3) | 60.200 | 136.161 | 60.171 | 136.186 |
| density (g/cm^3) | 5.062 | 3.898 | 5.060 | 3.897 |
| Crystallite size (mm) | 0.190 | 0.012 | 0.131 | 0.005 |
| Rw factor | 0.23704 | 0.23704 | 0.13126 | 0.13126 |

**2. The Electron Selectivity Balance**

In order to better understand the reduction process and the distribution of the photogenerated charge carriers between gas and liquid phase, the electron selectivity was estimated based on the number of electrons transferred per molecule of each product. The electron-based selectivity to a certain phase or product is represented as [25]:

**e-Selectivity** $\boldsymbol{=}\frac{\sum\boldsymbol{(}\boldsymbol{e}_{\boldsymbol{x}}\boldsymbol{\times}\boldsymbol{n}_{\boldsymbol{x}}\boldsymbol{)}}{\sum\boldsymbol{(}\boldsymbol{e}_{\boldsymbol{i}}\boldsymbol{\times}\boldsymbol{n}_{\boldsymbol{i}}\boldsymbol{)}}$ $\boldsymbol{\times}$ **100%**

where **e_x_** and **n_x_** are the number of electrons required and the molar quantity for the specific target product (or phase) being evaluated. In the denominator, **e_i_** and **n_i_** represent the electron requirements and molar quantities for all detected products **i** in the system (2 for CO, 8 for CH_4_, 6 for CH_3_OH, and 12 for C_2_H_5_OH), measured at 4 hours:

**Total Electrons Consumed(** $\sum\boldsymbol{(}\boldsymbol{e}_{\boldsymbol{i}}\boldsymbol{\times}\boldsymbol{n}_{\boldsymbol{i}}\boldsymbol{)}$**):**

CO: (1.3µmol g^-1^ x 2e^‑^) = 2.6µmol e^‑^ g^-1^

CH_4_: (6.3µmol g^-1^ x 8e^‑^) = 50.4µmol e^‑^ g^-1^

Methanol: (66.33µmol g^-1^ x 6e^‑^) = 397.98µmol e^‑^ g^-1^

Ethanol: (44.21µmol g^-1^ x 12e^‑^) = 530.52µmol e^‑^ g^-1^

Total Electrons Consumed = 981.50µmol e**^‑^** g^-1^

**Final Phase Selectivity Calculation:**

Gas Selectivity = (53.0 / 981.50) x 100 = 5.4%

Liquid Selectivity = (928.50 / 981.50) x 100 = 94.6%

**3. Morphology of the STO and Cu:STO**

**Figure.** **S****1(a-c)** FESEM image and corresponding mapping analysis of STO sample**.**

**Figure. S2(a-d)** FESEM image and corresponding mapping analysis of Cu:STO sample**.**

Figure. S3 (a) Full-scan mass spectrum of the gaseous products. (b) Time-resolved online mass spectrometry (MS) signals monitoring ionic fragments of gaseous and volatile products during alternating light ON/OFF cycles.

**Table S2:** The percentage of the availability of Ti^3+^, Ti^4+^, and proportion of Ti^3+^/Ti^4+^ determined by XPS spectra after the CO_2_ photoreduction stabilty test.

| **Phases** | **%Ti^3+^** | **%Ti^4+^** | **Ti^3+^/Ti^4+^** |
| --- | --- | --- | --- |
| ***SL*** | **44.84** | **55.16** | **0.813** |
| ***SG*** | **61.56** | **38.44** | **1.6** |
| ***PSG*** | **69.73** | **30.27** | **2.3** |

**References:**

1.	Hezam, A., Alkanad, K., Bajiri, M.A., Strunk, J., Takahashi, K., Drmosh, Q.A., Al‐Zaqri, N., and Krishnappagowda, L.N. (2023) 2D/1D MoS2/TiO2 heterostructure photocatalyst with a switchable CO2 reduction product. *Small methods*, **7** (1), 2201103.

2.	Alkanad, K., Hezam, A., Drmosh, Q.A., Ganganakatte Chandrashekar, S.S., AlObaid, A.A., Warad, I., Bajiri, M.A., and Neratur Krishnappagowda, L. (2021) Construction of Bi2S3/TiO2/MoS2 S‐scheme heterostructure with a switchable charge migration pathway for selective CO2 reduction. *Sol. RRL*, **5** (11), 2100501.

3.	Luo, C., Zhao, J., Li, Y., Zhao, W., Zeng, Y., and Wang, C. (2018) Photocatalytic CO2 reduction over SrTiO3: Correlation between surface structure and activity. *Appl. Surf. Sci.*, **447**, 627–635.

4.	Shao, K., Wang, Y., Iqbal, M., Lin, L., Wang, K., Zhang, X., He, M., and He, T. (2018) Modification of Ag nanoparticles on the surface of SrTiO3 particles and resultant influence on photoreduction of CO2. *Appl. Surf. Sci.*, **434**, 717–724.

5.	Zeng, S., Kar, P., Thakur, U.K., and Shankar, K. (2018) A review on photocatalytic CO2 reduction using perovskite oxide nanomaterials. *Nanotechnology*, **29** (5), 52001.

6.	Takata, T., and Domen, K. (2009) Defect engineering of photocatalysts by doping of aliovalent metal cations for efficient water splitting. *J. Phys. Chem. C*, **113** (45), 19386–19388.

7.	Łącz, A., Gwóźdź, P., Mizera, A., Górecka, S., Pacultová, K., Obalová, L., Górecki, K., Piech, R., Kramek, A., and Drożdż, E. (2024) Cu and Co-modified SrTiO3 as materials for environmental applications. *Surfaces and Interfaces*, **44**, 103672.

8.	Xu, Y., Liang, Y., He, Q., Xu, R., Chen, D., Xu, X., and Hu, H. (2022) Review of doping SrTiO3 for photocatalytic applications. *Bull. Mater. Sci.*, **46** (1), 6.

9.	Ding, J., Qiu, C., Zhang, Z., Qu, Q., Fang, C., Zhang, J., Guan, G., and Wan, H. (2024) Enhanced photocatalytic CO2 reduction to methanol by modulating valence states of copper in CuSrTiO3. *Sep. Purif. Technol.*, **349**, 127876.

10.	Sun, H., Dong, C., Huang, A., Zhan, H., Wang, G., Liu, W., Ma, B., and Wang, W. (2022) Transition Metal Doping Induces Ti3+ to Promote the Performance of SrTiO3@TiO2 Visible Light Photocatalytic Reduction of CO2 to Prepare C1 Product. *Chem. – A Eur. J.*, **28** (28), e202200019.

11.	Li, Y., Zhang, Y., Shi, L., Liu, X., Zhang, Z., Xie, M., Dong, Y., Jiang, H., Zhu, Y., and Zhu, J. (2024) Activating Inert Perovskite Oxides for CO2 Electroreduction via Slight Cu2+ Doping in B-Sites. *Small*, **20** (37), 2402823.

12.	Inoue, T., Fujishima, A., Konishi, S., and Honda, K. (1979) Photoelectrocatalytic reduction of carbon dioxide in aqueous suspensions of semiconductor powders. *Nature*, **277** (5698), 637–638.

13.	Li, Y., Wang, W.-N., Zhan, Z., Woo, M.-H., Wu, C.-Y., and Biswas, P. (2010) Photocatalytic reduction of CO2 with H2O on mesoporous silica supported Cu/TiO2 catalysts. *Appl. Catal. B Environ.*, **100** (1–2), 386–392.

14.	Ola, O., and Maroto-Valer, M.M. (2014) Role of catalyst carriers in CO2 photoreduction over nanocrystalline nickel loaded TiO2-based photocatalysts. *J. Catal.*, **309**, 300–308.

15.	Bajiri, M.A.M., Khan, N., Albornoz Diaz, J.C.C., Avansi Jr, W., Gouvêa, D., and Gonçalves, R. V (2025) Engineering Stable Cu-Doped SrTiO3 Perovskites for Enhanced Photocatalytic CO2 Reduction. *Inorg. Chem.*

16.	Han, T., Chen, Y., Tian, G., Zhou, W., Xiao, Y., Li, J., and Fu, H. (2016) Hydrogenated TiO 2/SrTiO 3 porous microspheres with tunable band structure for solar-light photocatalytic H 2 and O 2 evolution. *Sci. China Mater.*, **59**, 1003–1016.

17.	Rufai, Y., Chandren, S., and Basar, N. (2020) Influence of solvents’ polarity on the physicochemical properties and photocatalytic activity of titania synthesized using deinbollia Pinnata leaves. *Front. Chem.*, **8**, 597980.

18.	Navas, J., Sánchez-Coronilla, A., Aguilar, T., Hernández, N.C., de los Santos, D.M., Sánchez-Márquez, J., Zorrilla, D., Fernández-Lorenzo, C., Alcántara, R., and Martín-Calleja, J. (2014) Experimental and theoretical study of the electronic properties of Cu-doped anatase TiO 2. *Phys. Chem. Chem. Phys.*, **16** (8), 3835–3845.

19.	Chen, J., Qiu, H., Zhao, Y., Yang, H., Fan, L., Liu, Z., Xi, S., Zheng, G., Chen, J., and Chen, L. (2024) Selective and stable CO2 electroreduction at high rates via control of local H2O/CO2 ratio. *Nat. Commun.*, **15** (1), 5893.

20.	Liu, L., Li, Y., He, J., Wang, Q., Deng, J., Chen, X., and Yu, C. (2024) Progress on gas-solid phase photoreactor and its application in CO2 reduction. *Green Chem. Eng.*, **5** (3), 290–306.

21.	Olivo, A., Ghedini, E., Signoretto, M., Compagnoni, M., and Rossetti, I. (2017) Liquid vs. Gas Phase CO2 photoreduction process: Which is the effect of the reaction medium? *Energies*, **10** (9), 1394.

22.	Ali, S., Flores, M.C., Razzaq, A., Sorcar, S., Hiragond, C.B., Kim, H.R., Park, Y.H., Hwang, Y., Kim, H.S., and Kim, H. (2019) Gas phase photocatalytic CO2 reduction,“A brief overview for benchmarking.” *Catalysts*, **9** (9), 727.

23.	Chen, Y., Jiang, L., Lin, S., Dong, P., Fu, X., Wang, Y., Liu, Q., and Wu, M. (2024) Carbon-Supported Fe-Based Catalyst for Thermal-Catalytic CO2 Hydrogenation into C2+ Alcohols: The Effect of Carbon Support Porosity on Catalytic Performance. *Molecules*, **29** (19), 4628.

24.	Sun, H., Sun, S., Liu, T., Zeng, J., Wang, Y., Yan, Z., and Wu, C. (2024) Integrated CO2 capture and utilization: selection, matching, and interactions between adsorption and catalytic sites. *Acs Catal.*, **14** (20), 15572–15589.

25.	Liu, S., Meng, Y., Chen, Z., Yan, J., Gao, F., Wu, T., and Yu, G. (2025) Cu9S5/Gel-Derived TiO2 Composites for Efficient CO2 Adsorption and Conversion. *Gels*, **11** (9), 711.

26.	Hiragond, C.B., Powar, N.S., Kim, H., and In, S.-I. (2024) Unlocking solar energy: photocatalysts design for tuning the CO2 conversion into high-value (C2+) solar fuels. *EnergyChem*, **6** (5), 100130.

27.	Li, D., Hao, C., Liu, H., Zhang, R., Li, Y., Guo, J., Vilancuo, C.C., and Guo, J. (2022) Photocatalytic CO2 conversion to ethanol: a concise review. *Catalysts*, **12** (12), 1549.

28.	Wang, Y., Chen, E., and Tang, J. (2022) Insight on reaction pathways of photocatalytic CO2 conversion. *ACS Catal.*, **12** (12), 7300–7316.

29.	Wang, Y., Liu, Y., Wang, L., Perumal, S., Wang, H., Ko, H., Dong, C.-L., Zhang, P., Wang, S., and Nga, T.T.T. (2024) Coupling photocatalytic CO2 reduction and CH3OH oxidation for selective dimethoxymethane production. *Nat. Commun.*, **15** (1), 6047.

30.	Zhang, A., Liang, Y., Li, H., Zhao, X., Chen, Y., Zhang, B., Zhu, W., and Zeng, J. (2019) Harmonizing the electronic structures of the adsorbate and catalysts for efficient CO2 reduction. *Nano Lett.*, **19** (9), 6547–6553.

31.	Wu, Z.-Z., Gao, F.-Y., and Gao, M.-R. (2021) Regulating the oxidation state of nanomaterials for electrocatalytic CO 2 reduction. *Energy Environ. Sci.*, **14** (3), 1121–1139.

32.	Kim, D., Lim, H., Seo, M., Shin, H., Kim, K., Jung, M., Jang, S., Chae, B., Park, B., and Lee, J. (2024) Study of CO2 adsorption properties on the SrTiO3 (001) surface with ambient pressure XPS. *ACS Appl. Mater. Interfaces*, **16** (29), 38679–38689.

33.	Nørskov, J.K., Bligaard, T., Rossmeisl, J., and Christensen, C.H. (2009) Towards the computational design of solid catalysts. *Nat. Chem.*, **1** (1), 37–46.

34.	Powar, N.S., Kwon, S., Hiragond, C.B., Lee, J., Gong, E., Kim, H.S., Kim, D., Goddard III, W.A., and In, S.-I. (2025) Defect-Driven Dynamics in Gas-Phase Photocatalytic CO2 Conversion to Solar Fuels Using Ti3+/Ti4+ Containing TiO2 and Nonstoichiometric Ag2S Nanowires. *ACS Catal.*, **15** (21), 18474–18483.
